# Comprehensive evaluation of disease coding quality in gastroenterology and its impact on the diagnosis-related group system: a cross-sectional study

**DOI:** 10.1186/s12913-023-10299-9

**Published:** 2023-12-21

**Authors:** Baiyang Yuan, Lili Quan

**Affiliations:** 1 Department of Medical Record Statistics Section, Anhui No.2 Provincial People’s Hospital, Hefei, Anhui China; 2 School of Public Health, Anhui Medical College, Hefei, Anhui China

**Keywords:** Gastroenterology, Disease diagnosis, Procedure coding, Improvement measures, Coding deficiencies, Diagnosis-related group, Performance

## Abstract

**Objective:**

According to the diagnosis-related group (DRG) requirement, issues of diagnosis and procedure coding in the gastroenterology department of our hospital were analyzed and improvement plans were proposed to lay the foundation for effective implementation of DRGs.

**Methods:**

The title page of case-history of 1600 patients admitted to the Department of Gastroenterology of this hospital from January 1, 2021 to December 31, 2021 was sampled as a data source, and the primary and other diagnostic codes, operation or procedure codes involved in the title page of case-history were categorized and statistically analyzed.

**Results:**

Of the 531 cases treated with gastrointestinal endoscopy in our hospital in 2021, coding errors were identified in 66 cases and unsuccessful DRG enrollment in 35 cases, including 14 cases with incorrect coding of the primary diagnosis (8 cases with unsuccessful DRG enrollment), 37 cases with incorrect coding of the primary operation (23 cases with unsuccessful DRG enrollment), and 8 cases with incorrect coding of both the primary diagnosis and the primary operation (4 cases with unsuccessful DRG enrollment). Analysis of 66 inpatient cases with coding problems showed a total of 167 deficiencies, including 36 deficiencies in major diagnoses, 84 deficiencies in other diagnoses, and 47 deficiencies in surgery or operation coding.

**Conclusion:**

The accuracy of coding of disease diagnosis and surgical operation is the basis for the smooth implementation of DRGs. The medical staff of this hospital has poor cognition of DRGs coding and fails to recognize the important role of the title page of case-history quality to DRGs system, and their attention to DRGs and knowledge base of disease classification coding should be improved. In addition, the high incidence of coding errors, especially the omission of disease diagnosis, requires increased training of physicians and nurses on clinical knowledge and requirements for DRGs medical records, thereby improving the quality of medical cases and ensuring the accuracy of DRGs information.

**Supplementary Information:**

The online version contains supplementary material available at 10.1186/s12913-023-10299-9.

## Introduction

The government health expenditure in China has been increasing year by year, but the people’s burden of medical care is still heavy [1]. Insufficient financial compensation for health from the government in China results in difficulties in sustaining the healthy and sustainable development of public hospitals. Due to the lack of government input, some hospitals neglect the social and public welfare of public hospitals to pursue economic benefits, and hospital/doctor income is linked to drugs and examination items, and doctors increase hospital income by indiscriminately prescribing high-priced drugs and increasing service items, which damages the interests of patients and the credibility of the medical profession, causing a crisis of trust between doctors and patients [2, 3]. A new system is urgently needed to reduce inflated drug prices, reduce unnecessary medical tests, and monitor medical practices, thereby reducing the burden of access to care and re-establishing trust between doctors and patients [4]. The medical payment method affects the supply-side behavior of medical services and can promote or constrain management efficiency, medical quality, resource utilization, and cost reimbursement. At present, the main payment methods used in China are post-payment systems such as payment by service items and payment by disease type and pre-payment systems such as capitation and total pre-payment. The post-payment system of medical expenses has simple operations but high management costs, and hospitals are prone to pursue economic efficiency and cause unnecessary consumption of resources, resulting in poor assurance of medical quality [5, 6].

The quality of inpatient title page of case-history data is an important aspect of medical quality management and is the main indicator of hospital medical quality and hospital management level. The development of medical cost payment from post-payment system to pre-payment system has become the development direction of the international medical insurance payment system [7, 8]. In response to the above problems, the diagnosis-related group (DRG) system is employed internationally to realize control of the unreasonable increase in medical costs and to guarantee the quality of medical services. DRGs are available for payment management, budget management, and quality of care management. It classifies different cases into several diagnostic groups for medical management based on the main diagnosis of the discharge title page of case-history, with comprehensive consideration of medical-related factors such as surgical operations, comorbidities, complications, age, severity of disease and regression, and resource consumption [9, 10]. The title page of case-history data completeness and accuracy is the prerequisite and foundation for the smooth implementation of DRGs, and standardization of disease coding standards and improvement of coding accuracy are the keys to effective grouping and medical management.

Our research team operates as a provincial quality control center for medical cases, providing us with specialized expertise in healthcare quality assessment. Furthermore, gastroenterology is of significant importance in our province due to its high prevalence and impact on public health. It ranks among the key development disciplines of clinical medicine in our region, with notable factors such as a high incidence rate and substantial healthcare costs associated with gastrointestinal diseases [11]. Therefore, this study analyzed the common problems and consequences of disease coding in gastroenterology from the perspective of the basic theory and principles of DRGs, and proposed improvement measures to improve the quality of disease coding and further promote the development of gastroenterology [12].

## Materials and methods

### Patient profiles

**Fig. 1:**
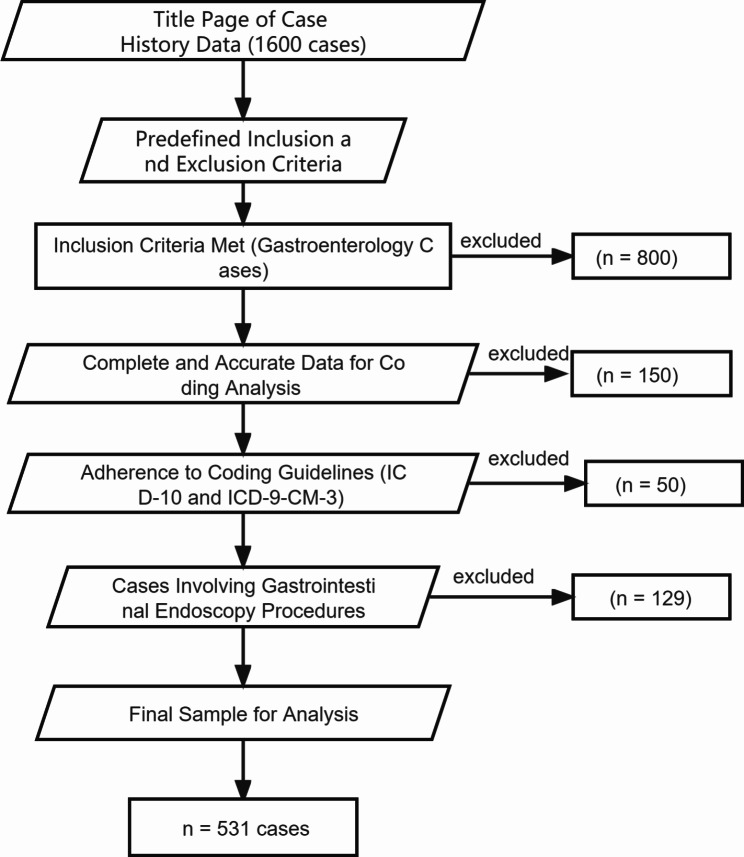
Flowchart of Participant Selection for Comprehensive Analysis of Disease Coding Quality in Gastroenterology

The title page of case-history of patients admitted to gastroenterology for treatment from January 1, 2021, to December 31, 2021, was extracted from the electronic medical record management system as the data source, and a total of 1600 medical records were collected. Based on the predefined inclusion and exclusion criteria, a total of 531 cases were ultimately included in the study for analysis (Fig. 1).

#### Inclusion criteria

(1) Gastroenterology Cases: Only cases related to gastroenterology were included in the study to maintain a specific focus on this medical discipline; (2) Complete and Accurate Data: Cases with missing or incomplete data related to disease diagnosis and surgical operation classification codes were excluded to ensure the accuracy and reliability of the coding analysis. (3) Adherence to Coding Guidelines: Cases that did not comply with the National Health Construction Commission’s Quality Specification for Filling in the Inpatient Title Page of Case-History and the clinical coding requirements of ICD-10 and ICD-9-CM-3 were excluded from the analysis. (4) Gastrointestinal Endoscopy Procedures: The study specifically aimed to analyze the quality of disease coding for gastrointestinal endoscopy procedures. Therefore, only cases involving gastrointestinal endoscopy were included in the final sample.

### Study indices

#### Methodology

The data for this study were derived from the title page of case-history, and the primary basis for grouping was the disease diagnosis and surgical operation classification codes using the DRGs system. The study aimed to conduct an in-depth analysis of case coding accuracy based on the requirements of the National Health Construction Commission’s Quality Specification for Filling in the Inpatient Title Page of Case-History and the national clinical 2.0 version of ICD-10 and ICD-9-CM-3 requirements.

#### Participant selection

To ensure comprehensive insights into the factors affecting coding quality, a purposive sampling approach was employed to select participants. The study included three groups of participants: medical and nursing staff (12 participants), case coders (5 participants), and case managers (3 participants). The selection criteria for medical and nursing staff were based on their expertise and experience in clinical care, medical coding, and hospital management. Certified case coders with experience in the application of ICD-10 and ICD-9-CM-3 coding systems were chosen. Case managers responsible for overseeing the inpatient title page of case-history and coding processes were included to provide insights into the workflow and management aspects.

#### Interview design and method

Semi-structured in-person interviews were conducted to explore the factors influencing coding quality (Appendix). The interview questions were designed to cover key areas such as coding guidelines and compliance, workflow and process, training and skill development, as well as challenges and solutions. Participants were asked about their understanding of the National Health Construction Commission’s Quality Specification for Filling in the Inpatient Title Page of Case-History and the clinical coding requirements of ICD-10 and ICD-9-CM-3. Emphasis was placed on exploring any challenges or ambiguities in the guidelines and their impact on coding accuracy. Additionally, participants were questioned about the coding process, roles and responsibilities of different stakeholders involved, potential areas of miscommunication or inefficiencies, and the training they received to enhance coding skills. They were also asked to identify major challenges during the coding process and propose potential solutions to address them effectively. The interviews were audio-recorded with the consent of the participants to ensure accurate capturing of information.

### Data statistical analyses

The data collected from the interviews were transcribed verbatim and analyzed using thematic content analysis. Excel software was utilized for data organization, summarization, and categorization. Major diagnosis coding, other diagnosis coding, operation and procedure coding data were analyzed using descriptive statistics to understand patterns and potential areas of improvement. To provide a measure of uncertainty and interpret the reported results at the population level, we have included a 95% confidence interval for relevant statistical measures (Table 1).

**Table 1:** Data requirements for DRGs grouping

Classification	Information/Data
Disease severity and complexity	Major diagnoses, comorbidities and complications, individual characteristics (sex, age, birth weight, days of life, etc.)
Medical need and intensity of use	Operating room procedures, non-operating room procedures, other ancillary medical and nursing services (e.g., ventilator use, etc.)
Medical outcomes	Discharge status (death, medical discharge, non-medical discharge, hospital referral)
Resource consumption	Medical costs, hospital days, cost classification
Coding system	ICD-10 is the diagnosis code; ICD-9-CM is the surgical procedure code
Data Sources	Inpatient title page of case-history

## Results

### Coding errors

Of the 531 cases treated with gastrointestinal endoscopy in our hospital in 2021, coding errors were identified in 66 cases and unsuccessful DRG enrollment in 55 cases, including 14 cases with incorrect coding of the primary diagnosis (8 cases with unsuccessful DRG enrollment), 37 cases with incorrect coding of the primary operation (23 cases with unsuccessful DRG enrollment), and 8 cases with incorrect coding of both the primary diagnosis and the primary operation (4 cases with unsuccessful DRG enrollment). (Table 2)

**Table 2:** Classification of coding errors and the effect on DRG

Errors	Number of errors	Error rate/%	95% CI (Lower - Upper)	Number of unsuccessful enrollment cases	unsuccessful enrollment rate /%	95% CI (Lower - Upper)
Primary diagnosis error	14	2.64	(1.49–4.42)	8	57.14	(29.29–81.99)
Primary operation error	37	6.97	(5.00–9.36)	23	62.16	(47.68–74.74)
Primary diagnostic and operational errors	8	1.51	(0.76–2.84)	4	50.00	(18.01–81.99)
	66	11.12	(8.88–13.84)	35	53.03	(40.38–65.21)

### Specific content of case deficiencies

Analysis of the content of coding deficiencies in gastroenterology analyzed 66 inpatient cases with coding problems, and a total of 167 deficiencies were identified, including 36 deficiencies in major diagnoses, including an incorrect selection of major diagnoses, incorrect coding, and inconsistency with pathology results. There were 56 deficiencies in other diagnoses, including omission, incorrect coding, and no combined coding; there were 47 deficiencies in surgery or operation coding, including an incorrect selection of major surgery, incorrect coding, incorrect or omitted filling of other surgery or operation, and incorrect filling of surgery level, incision, and anesthesia mode. (Table 3)

**Table 3:** Specific content of coding deficiencies

Errors	Deficiencies	Number of deficiency/n	Composition ratio /%	95% CI (Lower - Upper)
Primary diagnosis	Incorrect selection	4	2.40	(0.77–6.06)
	Incorrect coding	15	8.98	(5.25–14.65)
	Inconsistent with pathology results	17	10.18	(6.17–15.82)
Other diagnoses	Missed coding	22	13.17	(8.40–19.32)
	Incorrect coding	24	14.37	(9.23–20.68)
	No combined coding	38	22.75	(16.71–29.73)
Surgery or operation	Incorrect selection of major procedure or operation	9	5.39	(2.61–10.34)
	Incorrect coding of major surgery or operation	18	10.78	(6.67–16.89)
	Other surgery or operation error or omission	6	3.59	(1.47–8.03)
	Incorrect filling of surgery or operation level, incision, anesthesia mode	14	8.38	(4.84–13.54)
	Total	167	100.00	

### Disease coding DRGs

With benign gastric tumors as an example, when coding different surgical procedures for the same disease, it was found that gastric lesion resection DRGs were categorized as GB1, while endoscopic submucosal dissection (ESD) was categorized as GK1. Combined with the presence of complications and comorbidities, DRGs were found to be grouped differently, which showed that the accuracy and refinement of disease and procedure coding are associated with DRGs. (Table 4)

**Table 4:** DRGs for different complications of different procedures for benign gastric tumors

Main diagnosis of disease	Major surgery	DRGs
Benign gastric tumor D13.100	Gastric lesion resection 43.4202	GB13 Major esophageal, gastric, and duodenal surgery with complications and comorbidities
		GB15 Major esophageal, gastric, and duodenal surgery without complications and comorbidities
	Endoscopic submucosal dissection of the stomach (ESD)43.4107	GK13Other endoscopic treatment operations of the digestive system with complications and comorbidities
		GK15Other endoscopic treatment operations of the digestive system without complications and comorbidities

### Effect of operation coding on DRG

The results of the present study show that operation coding has a greater impact on enrollment in cases treated by gastrointestinal endoscopy. The same diagnosis might have different operation codes, and miscoding or inaccurate coding could have implications for enrollment. There are six main operation coding error cases, and the DRG enrollment control before and after the operation coding correction was performed for each error case. (Table 5)

**Table 5:** DRG before and after correction of major operation coding errors

Before correction			After correction		
Primary operation name (Code)	Adjacent diagnosis related groups	Points	Primary operation name (Code)	Adjacent diagnosis related groups	Point
Fiberoptic colonoscopic colon polypectomy (45.4200 × 003)	GK3 Colonoscopic treatment operation	50.14	Endoscopic mucosal resection (EMR) (45.4307)	Gastrointestinal system other endoscopic treatment operations	77.81
Endoscopic sigmoid colon polypectomy (45.4201)	GK3 colonoscopy operation	50.14	Endoscopic resection of sigmoid colon lesions (45.4302)	Other operations of digestive system	145.11
Rectal [endoscopic] polypectomy (48.3600)	GZ1 Other digestive system diagnoses	39.37	Endoscopic mucosal resection (EMR) (48.3510)	GK1 Other endoscopic treatment operations of the digestive system	77.81
Endoscopic colorectal polypectomy (45.4200)	GZ1 Other digestive system diagnoses	39.37	Endoscopic submucosal dissection (ESD) (45.4300 × 009)	GK1 Other endoscopic treatment operations of the digestive system	77.81
Endoscopic colorectal polypectomy (45.4200)	GZ1 Other digestive system diagnoses	39.37	Colon polypectomy with fiberoptic colonoscopy (45.4200 × 003)	GK3 Colonoscopic treatment operation	50.14
Endoscopic colorectal polypectomy (45.4200)	GZ1 Other digestive system diagnoses	39.37	Endoscopic resection of colonic lesions (45.4302)	GK3 Colonoscopic treatment operation	50.14

## Discussion

DRGs are currently the most promising prepayment method for health insurance and are used as an important basis for hospital performance evaluation systems [13, 14]. The completeness and accuracy of the title page of case-history data and the rigor of the connotation quality of the original data of the case are critical to the results, which directly affect the DRGs and, consequently, the objectivity and accuracy of the hospital performance evaluation results based on DRGs [15–17]. Gastrointestinal tract tumors are a common oncological disease, and according to cancer center data, esophageal, gastric and liver cancers account for 25.94% and 34.43% of all new malignant tumors and death cases of malignancies [18, 19]. With the gradual popularization of the application of gastrointestinal endoscopy, endoscopic treatment techniques such as endoscopic high-frequency electrical polypectomy, endoscopic mucosal resection, and endoscopic mucosal peeling play an important role in the early diagnosis and treatment of early cancer and precancerous lesions of the gastrointestinal tract due to less trauma, shorter hospital stay, and lower cost versus open surgery. The correct coding of the diagnosis and operation of the title page of case-history is the key to fully reflect the skill and value of the physician’s treatment [20]. On title page of case-history data filling, the disease classification of patients with digestive diseases involves more coding principles, including at least 2 codes for site and morphology, which is the most difficult and most controversial Sect. [21]. It is instructive to evaluate the quality of the disease classification data of patients with digestive diseases to explore effective measures to improve the quality of title page of case-history data, which can provide data support for DRGs performance evaluation and payment method reform.

### Analysis of the causes of title page of case-history issues

#### Insufficient attention to medical record writing

Physicians should fulfill their responsibilities for medical cases and collect information about patients in an accurate, complete and timely manner. Some clinicians only focus on clinical treatment or surgery, with little demand for medical record writing, and they are not aware of the importance of the title page of case-history in the whole case. In addition, despite the establishment of a quality control system for archived cases, i.e., physicians, departmental quality controllers, and the hospital case management committee, all levels fail to give full play to their responsibilities. Due to the heavy workload, clinicians usually check the medical records roughly after completion and hand them over to the quality controllers, while some quality controllers fail to perform detailed quality control of the cases and only sign the record, and the case management committee fails to achieve full coverage of quality control of archived cases, leading to case-report deficiencies [22].

#### Insufficient familiarity with medical norms

Some physicians rarely attend medical record writing standard training, or pay little attention to the training, resulting in a limited understanding of filling in the title page of case-history. For example, some physicians fill in “none” for an item with no content, which, however, should be filled with “-“, or some give no consideration to the logic of the content, resulting in errors [23].

#### Inadequate implementation of training assessment

The training of medical record writing standards is routinely conducted once a year. However, many physicians are absent during the training, such as outpatient and inpatient on-call staff, emergency surgery staff, and emergency resuscitation staff. The absent physicians rarely study the training content after the training, and the departmental quality control staff fails to provide regular training on medical record writing standards to the departmental physicians. Moreover, the coding staff fails to understand the diagnosis filled out by the physician, resulting in incorrect or missed coding. The total score of the whole case-history is 100 points, and the title page of case-history score is only 5 points, and each item in the title page of case-history has a low value, leading to insufficient attention from physicians [24].

#### Inadequate electronic medical record information system

A logical data verification function is absent in the electronic medical record. The most serious deficit is that despite the existence of surgical or anesthesia fees, the title page of case-history is empty for surgery and operation, and no indications are prompted in the event of incorrect filling of the sex of patients. There are inconsistencies between the amount of total costs extracted from the cost patient in the electronic medical record and the costs derived from the HIS system [25].

### Summary of coding quality problems in gastroenterology cases and analysis of causes

#### Primary diagnostic deficiencies

The accuracy of the primary diagnosis deficiency, as the data basis for diagnosis-related classification, is the key to the effective implementation of diagnosis-related classification. (1) Clinicians have little knowledge of disease coding and have no conceptual understanding or unclear understanding of the primary diagnosis, which leads to frequent errors in the primary diagnosis in gastroenterology. (2) Lack of professional knowledge and work responsibility of coders is also one of the important reasons. Most of the coders have relatively little medical expertise, insufficient communication with clinicians, and inattentive reading of cases, resulting in a high propensity for coding errors. If coders have little professional medical knowledge and are not clear about the process of primary diagnosis, the following principles should be carefully considered: When a patient suddenly develops a more serious complication or other diseases during hospitalization than at the time of admission, the most serious disease should be used as the primary diagnosis. (3) The inconsistency between the primary diagnosis and the pathological findings generally emerges because the pathological diagnosis has not been sent to the gastroenterology department after the patient is discharged from the hospital, leading to the neglect of the primary diagnosis of a confirmed tumor [26, 27].

#### Other diagnostic deficiencies

Other diagnostic deficiencies in gastroenterology include incorrect coding, omission, and failure to use combined codes. For example, after the clinicians made the diagnosis of cholecystitis and gallbladder stones respectively, the coders did not timely combine the codes for gallbladder stones with cholecystitis and may have missed filling in other diagnoses due to the misuse of the combined codes. Incomplete clinical diagnosis and irregular diagnostic terminology are also one of the primary causes of other diagnostic defects. For example, the coder should have filled in acute severe pancreatitis as the other diagnosis of the discharged patient, but mistakenly filled in acute pancreatitis, resulting in coding errors [28].

#### Operational and surgical deficiencies

Gastroenterology cases have the highest incidence of operational and surgical deficiencies: (1) Clinicians fill out the electronic medical record without knowing the level of the procedure or fill it out only according to the date of the procedure instead of the coding rules, and the coder fails to timely correct the problem, leading to the incorrect selection of the primary procedure. (2) The treatment of gastroenterological diseases usually involves various endoscopic, gastroscopic, and enteroscopic operations, which may lead to coding errors. (3) Errors in the writing of healing grade, surgical incision grade, and failure to follow the anesthesia record sheet for anesthesia modality writing are also more common causes [29, 30].

### Case coding improvement measures

#### Enhanced communication between doctors and coders

Physicians and coders differ in their understanding of disease classification due to their different areas of expertise. Therefore, strengthened communication and collaboration between the two to jointly promote the quality of case management is effective to ensure the accuracy and consistency of coding.

#### Improvement of medical record quality and clinical expertise

Accurate completion of the discharge diagnosis is the key to medical case writing, which centralizes all disease types of patients and also has a direct impact on both statistical accuracy and coding quality of medical data. In addition, the coders’ lack of clinical expertise and over-reliance on the coding library will compromise the efficiency and quality of coding. Therefore, it is necessary to strengthen pre-service medical record writing training for new doctors, hold regular special seminars on medical record writing, conduct regular random checks on medical case writing by the medical department and link it to performance assessment, set up a case review room to strictly check the final medical records, carry out regular clinical medical knowledge training for coders, so as to reduce the cases of omission and misfiling.

#### Enhancement of patient case management

Disease coding, as the main basis for payment by disease at this stage, plays an important role in improving the overall service quality of hospitals. Therefore, the coding work and medical record writing process need to be further standardized and managed, and the promotion of titles should be correlated with the quality of medical record writing. The principles of coding use, such as surgical coding and International Classification of Diseases standard coding, were fully implemented, and regular training was performed to reduce the coding errors.

The sample size of this questionnaire is small, and the data findings may be biased. The questionnaire items were superficial, and the case coding quality study was unable to regroup incorrect codes due to certain constraints. Future studies will increase the sample size, modify the questionnaire items, obtain a deeper understanding of the medical staff, and investigate in depth the impact of incorrect coding on DRGs grouping and the impact on hospital medical costs, so as to increase the attention of hospital medical staff to the quality of coding.

## Conclusion

The prominent problem of case coding in our gastroenterology department is case coding errors, and the main factor that affects the quality of case coding is operation or procedure coding defects, followed by major diagnostic defects. It is essential to adopt measures for the standardization of medical record writing, strengthen the control of title-page coding quality, and circumvent the problem of coding defects in medical cases so as to reduce the incidence of diagnostic errors. The principles of coding use, such as surgical coding and International Classification of Diseases standard coding, should be fully implemented and the relevant personnel should be trained regularly to master the coding rules in order to reduce the incidence of coding errors. Besides coding errors, inadequate review of DRGs could also be responsible for inappropriate financing. Regular review and correction of cost factors are also crucial. There are a few limitations that need to be addressed. First, this study only examined disease coding practices in a single institution and department. While this allowed us to conduct an in-depth analysis with our specific expertise and access to data, it may limit the generalizability of the findings to other healthcare settings. Future research involving multiple institutions and departments would be valuable to obtain a more comprehensive view of disease coding practices in the field of gastroenterology.

### Electronic supplementary material

Below is the link to the electronic supplementary material.

Supplementary Material 1

## Data Availability

All data generated or analysed during this study are included in this published article.
