# Supplementary material for: Comprehensive evaluation of disease coding quality in gastroenterology and its impact on the diagnosis-related group system: a cross-sectional study
Source: BMC Health Serv Res. 2023 Dec 21;23:1451. doi: 10.1186/s12913-023-10299-9 (PMC10740297; doi:10.1186/s12913-023-10299-9)
Supplement: Supplementary file 1 — Supplementary Material 1 [file 12913_2023_10299_MOESM1_ESM.docx]

Here are some interview questions in a Semi-structured in-person interviews:

**Part 1: Personal Background**

1. Your specialty:

A. Internal Medicine

B. Surgery

C. Gynecology

D. Obstetrics

E. Neonatology

F. Pediatrics

G. Rehabilitation Medicine

H. Other

1. Your current professional title:

A. Chief Physician

B. Associate Chief Physician

C. Attending Physician

D. Resident Physician

E. Assistant Physician

1. Years of professional experience:

A. Less than 3 years

B. 3-5 years

C. 5-10 years

D. More than 10 years

1. Your highest educational qualification:

A. Vocational/Technical

B. Bachelor's

C. Master's

D. Doctorate

**Part 2: Departmental Medical Record Management**

1. Does your department have established medical record management procedures and processes?

A. Yes

B. No

C. I'm not aware

1. Your familiarity with your department's medical record management procedures:

A. Very familiar, knowledgeable about the entire process

B. Familiar with my responsibilities in the process

C. Unfamiliar and not involved

1. Are the department's medical record management procedures linked to individual performance assessments?

A. Yes, strictly enforced

B. Yes, but not strictly enforced

C. No

1. What is the required 3-day filing rate for discharge summaries in your department?

A. 80%

B. 90%

C. 95%

D. 100%

1. Your understanding of the process for correcting errors in inpatient records:

A. Very familiar, have made corrections

B. Familiar, but haven't made corrections

C. Unfamiliar

1. Your understanding of the process for correcting errors in archived discharge records:

A. Very familiar, have made corrections

B. Familiar, but haven't made corrections

C. Unfamiliar

1. Your knowledge of the procedure for clinical departments to request, copy, or review medical records at the medical record management department:

A. Very familiar

B. Partially familiar

C. Completely unfamiliar

**Part 3: Training on Medical Record Front Page Coding**

1. If you were to rank the importance of various components of a medical record, what would be the most important?

A. Front Page

B. Admission Records

C. Progress Notes

D. Discharge Records

E. Informed Consent and Disclosure Forms

F. Operative Reports

1. Within the front page of a medical record, which component do you consider most important?

A. Patient Demographics

B. Inpatient Progress Information

C. Diagnostic and Treatment Information

D. Billing Information

1. The primary diagnosis code and primary procedure code are part of which section on the medical record front page?

A. Patient Demographics

B. Inpatient Progress Information

C. Diagnostic and Treatment Information

D. Billing Information

1. General principles for selecting the primary diagnosis code:

A. The diagnosis with the greatest impact on the patient's health, resource utilization, and length of stay.

B. The diagnosis with the highest resource utilization, the greatest impact on the patient's health, and the longest length of stay.

C. The diagnosis with the longest length of stay, the highest resource utilization, and the greatest impact on the patient's health.

1. Have you studied the "National Health Construction Commission's Quality Specification for Filling in the Inpatient Title Page of Case-History"?

A. Yes

B. No

1. How did you learn about the "National Health Construction Commission's Quality Specification for Filling in the Inpatient Title Page of Case-History"?

A. Hospital-level training

B. Rotations in the Medical Record Department

C. Hospital's distribution of the document

D. Attending academic conferences

E. Online learning

F. Not attended

1. Did you participate in the hospital's "Sub-department Coding Training" on medical record front page coding?

A. Yes

B. No

1. Which form of training for medical record front page coding do you find most effective?

A. Centralized training by the Medical Record Management Department in the sub-department

B. Training of sub-department coding quality control personnel by the Medical Record Management Department, followed by sub-department training

C. Hospital-wide collective training

D. Online training via platforms such as WeChat or the hospital's intranet

1. Rate your level of acceptance of the hospital's "Sub-department Coding Training" on medical record front page coding:

A. 20-30%

B. 50-60%

C. 70-80%

D. 90-100%

1. How would you evaluate the effectiveness of the hospital's "Sub-department Coding Training" on medical record front page coding?

A. Very satisfied

B. Somewhat satisfied

C. Not satisfied

1. Do you think your department needs to conduct further training on medical record front page coding?

A. Very necessary

B. Necessary

C. Indifferent

D. Not necessary

1. How frequently do you believe medical record front page coding training should be conducted?

A. Monthly

B. Quarterly

C. Semi-annually

D. Annually

1. What is an appropriate duration for a single medical record front page coding training session?

A. 30 minutes

B. 40 minutes

C. 60 minutes

D. 90 minutes

1. When the Medical Record Department requests your assistance to improve front page coding, how do you respond?

A. Willing and immediate action

B. Willing but not enthusiastic

C. Consider it a hassle and avoid it

1. How does your department handle end-of-stay medical record quality control?

A. Conduct quality control for every case

B. Partial quality control

C. No quality control

D. Selectively based on workload

1. When your department identifies issues with the front page of a medical record, how is the feedback communicated to you?

A. Departmental meetings

B. Departmental quality control meetings

C. Direct one-on-one communication

D. Morning handovers
